# Wastophobia: the driver of e-waste management - antecedents and consequences

**DOI:** 10.3389/fpsyg.2025.1537410

**Published:** 2025-07-09

**Authors:** Muhammad Wasif Hanif, Muhammad Nawaz, Hui Wang

**Affiliations:** ^1^College of Economics and Management, Hanshan Normal University, Chaozhou, China; ^2^College of Economics and Management, Nanjing University of Aeronautics and Astronautics, Nanjing, China; ^3^Urban Construction Project Management Center, Zaozhuang Housing and Urban Rural Development Bureau, Zaozhuang, China

**Keywords:** awareness of consequences, creative performance, moral courage, pro-environmental behavior, wastophobia

## Abstract

The rapid growth of technological advancements is boosting planned obsolescence behavior, subsequently reducing the lifecycle of electronic products, and raising electronic waste (e-waste) concerns globally. Considering this dilemma, this study aims to explore the antecedents and consequences of wastophobia to promote sustainable consumption behavior, mitigate e-waste, and enhance environmental performance. Data were collected from the electronics industry consumers (*n* = 302) and analyzed through structural equation modeling via SPSS and AMOS-26. The results found two fundamental antecedents of wastophobia, including awareness of wasteful consumption and awareness of consequences, which are interrelated but distinct constructs. Together, these determinants significantly cultivated wastophobia in consumer behavior. Moreover, heightened wastophobia has impacted significantly positively on multiple behavioral outcomes, including creative performance, moral courage, and pro-environmental behavior (except for consumer advocacy). The elevated wastophobia rooted in emotions, such as dismay, culpability, and decrepit significantly improves the usability cycle of products, reduces planned obsolescence, e-waste, and consequently enhances environmental performance. This study suggests stakeholders (consumers, organizations, governments, and society) to promote wastophobia culture at societal (community and organizations), national, and global levels to minimize e-waste.

## 1 Introduction

**Figure 1:**
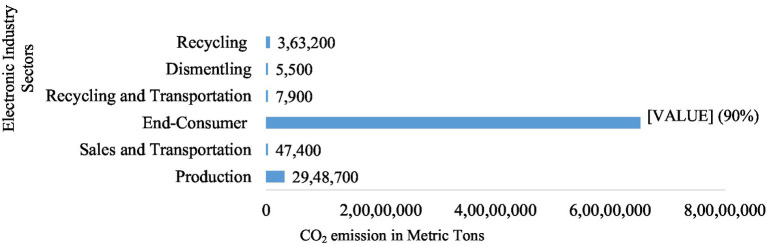
Electronic industry CO2 emissions in China (Wang et al., 2024).

There is no doubt that technology has made significant progress over the years but the irony is that such improvements are increasing the inefficiency in resource consumption and polluting the environment. For example, the electronic products manufacturing sector in China, which deals with the highest number of electronic goods globally, often presents new products with minimal improvements in functionality but have fascinating changes in design. This trend contradicts the theory of responsible consumption (Fisk, 1973) since it encourages people to dispose of functioning appliances for newer versions, portraying why around 6 billion functional mobile phones will be discarded in China by the end of 2025 (Liu et al., 2023). Thus, it is not surprising that around 20 percent of the e-waste from the world comes from China, increasing yearly by 3 to 5% (Wang et al., 2024). This pattern of consumption contributes to 90% of CO2 emissions (see Figure 1) (Wang et al., 2024) reduces product life cycles (Yu et al., 2023), and promotes planned obsolescence (Alzaydi, 2024). These consumer consumption behavior models are detrimental to the efforts of researchers and policymakers struggling for sustainable development (Roberts et al., 2023).

The inefficient consumption pattern nurtures the culture of disposability at the cost of the environment. Alarmingly, only 17% of the total global e-waste is recycled appropriately. The rest is dumped into landfills (Sherif et al., 2024), which causes the release of harmful substances like cadmium, mercury, and lead (Arya et al., 2023), and turns healthy land, environment, and water sources into hazardous sites. Past researchers efficiently utilized numerous behavior strategies to promote sustainable consumption behavior such as electricity prices (Chen, 2017), energy taxes (Mills and Scheleich, 2010), the role of incentives (Edelman, 2015), and education (Nawaz et al., 2022; Khan et al., 2018). However, the ratio of e-waste has persistently risen over time. However, the ratio of e-waste has persistently risen over time. It is reasonable to believe that the globe is at a juncture of immense pressures, including the frequent discard of functional appliances, intentions of planned obsolescence (short product lifecycle), recycling challenges, and rising environmental concerns, with no fundamental solution in hand.

To the best of the researcher’s knowledge, there is a notable gap in the literature exploring the potential role of wastophobia on sustainable consumption behavior to manage rising trends in e-waste. Wastophobia is defined as *a state of fear that promotes considerate behavior and deters the way an individual develops mental precociousness, apprehension, and the practices of wasteful consumption. It may give rise to dismay and feel individual culprit, decrepit, and accountable for wasteful consumption practices that are inconsiderate*” (Hanif et al., 2022, p. 271). Research suggests that consumers with a high level of wastophobia are more likely to modify their consumption pattern, ultimately maintaining sustainable consumption behavior over time (Nawaz et al., 2025).

**Figure 2:**
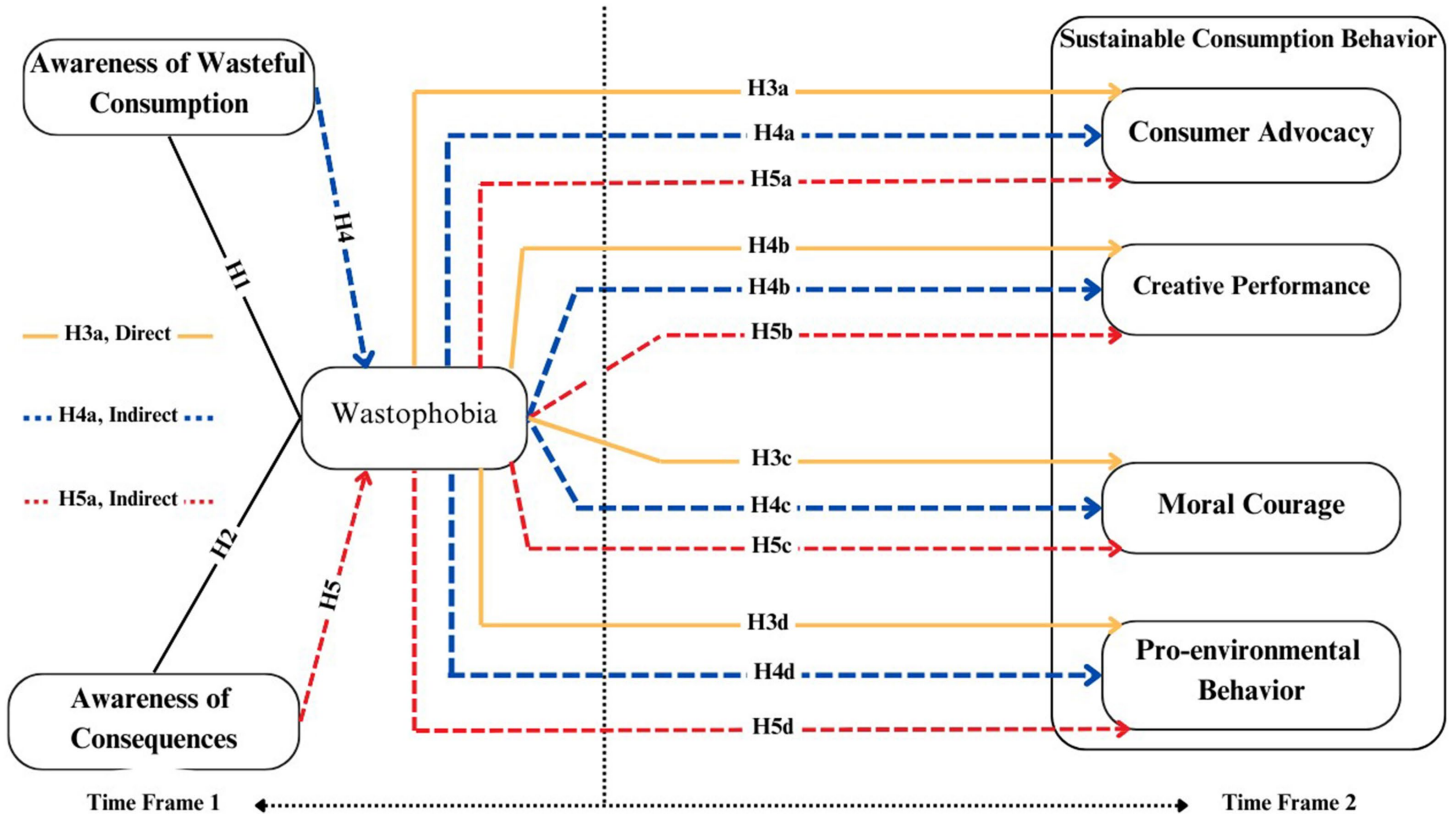
Research framework.

In this era of paradoxical technological advancement, where inventions often exacerbate e-waste, the concept of wastophobia can serve as a powerful psychological catalyst. Understanding the concept of wastophobia and its relationship with different behavioral aspects (as depicted in Figure 2) could yield new insights into consumer behavior and psychology. Additionally, none of the studies have explored the fundamental antecedents and consequences of wastophobia. Insights into these aspects could enable researchers, policymakers, and governments to promote a wastophobia culture, *characterized by an environment that pampers individuals into heightened anxiety about wasteful consumption within society and organizations*. The exploration of antecedents and consequences of wastophobia can provide deeper insights into comprehensively controlling e-waste.

Therefore, the present study proposes two dimensions of consumer awareness: including (a) awareness of wasteful consumption and (b) awareness of consequences that can catalyze sustainable consumption behavior. Awareness plays a crucial role in promoting sustainable consumption habits (Hanif et al., 2021). However, past research has largely overlooked the combined effect of awareness of wasteful consumption and awareness of consequences regarding wastophobia. This study suggests that when these dimensions are combined, they can produce the phenomenon of wastophobia in the minds of consumers. When activated, this wastophobia—embedded in persistent feelings of fear, shame, and guilt can trigger various behavioral consequences, including consumer advocacy, creative performance, moral courage, and pro-environmental behavior. The selection of these variables is grounded in prior literature that portraying the significant role of awareness of wasteful consumption (Hanif et al., 2021), awareness of consequences (Schwartz, 1977), consumer advocacy (Hamby et al., 2024), creative performance (Sabokro et al., 2021), moral courage (Kemper et al., 2023), and pro-environmental behavior (Parvatiyar and Sheth, 2023) in shaping consumer behavior. However, the interaction of wastophobia within the context of e-waste management has remained largely ignored. To address these concerns in the literature and the marketplace, this study formulates two research questions to enhance the comprehension of consumer behavior.

*RQ1: What are the antecedents that lead to wastophobia in consumer behavior?* Understanding the antecedents can help stakeholders cultivate a culture of wastophobia that discourages inefficient consumption practices.

*RQ2: What behavioral changes does wastophobia induce, and how does it help to distort the prevailing culture of disposability and unsustainable consumption practices?* Insights from these behavioral outcomes will support fostering sustainable consumption behavior.

This study establishes a theoretical framework centered on the wastophobia construct, illustrating how the antecedents of wastophobia—awareness of wasteful consumption and awareness of consequences—are interrelated yet distinct in activating wastophobia. The activation of wastophobia is expected to trigger a beneficial chain reaction of behavioral outcomes for environmental performance, including creative performance, moral courage, and pro-environmental behavior (excluding consumer advocacy). These outcomes motivate consumers to think critically and act sustainably, thereby reducing the frequent disposal of electronic appliances and minimizing e-waste. From the practical point of view, the study recommends implementing behavioral strategies to cultivate wastophobia at various levels: societal (community and organizational), national, and at global. This multipronged technique can help to reduce waste across different domains, including water, food, plastic, and particularly e-waste, by inspiring consumers to extend the usability of their resources. Moreover, a persuasive culture of wastophobia could pressure organizations to prioritize sustainable technological advancements over short-term profitability.

## 2 Review of the literature and hypotheses development

### 2.1 Awareness of wasteful consumption and wastophobia

Awareness of wasteful consumption refers to the understanding and recognizing excessive or inefficient use of resources (Hanif et al., 2021, p. 123), and significantly influences individuals’ emotions toward wasteful consumption practices (Hanif et al., 2022; Zhang et al., 2025). This awareness aligns with the conservation of resources theory, which suggests that individuals are inherently motivated to protect their physical, social, and psychological resources (Schwartz, 1977). A growing body of literature demonstrates that increased awareness triggers adverse emotional responses such as anxiety, stress, and fear (Subramanian et al., 2022). However, the literature does not significantly identify the role of recognizing one’s wasteful behaviors in the context of e-waste generation. It is reasonable to assume that heightened awareness of wasteful consumption practices can make consumers more concerned about their actions, thereby triggering anxiety, fear, or worry towards wastefulness—termed as wastophobia. Therefore, considering based on the claims of Subramanian et al. (2022), Hanif et al. (2022), and Schwartz (1977), this study assumes that awareness of wasteful consumption can significantly impact wastophobia.

*H_1_*: Awareness of wasteful consumption has a significant positive impact on wastophobia.

### 2.2 Awareness of consequences and wastophobia

Awareness of consequences is a cognitive state wherein individuals understand the consequential impacts of their actions on health, communities, and ecosystems (Ryan and Spash, 2012, p. 2509; Schwartz, 1977). According to norm activation theory, such awareness activates personal norms which not only activate ethical standards but also promote sustainable consumption behavior (Schwartz, 1977). This heightened awareness makes individuals sensitive toward their personal needs (Harland et al., 2007) and compels them to reassess the ramifications of actions affecting human health and the environmental (Wang et al., 2018). According to Ryan and Spash (2012), such consciousness is a fundamental pillar of ethical standards. A recent study of Badawi et al. (2024) supports this claim, portraying that consumer possessing heightened awareness of consequences are more likely to boost their environmental behavioral intentions regarding plastic waste management. Despite that awareness of consequences significantly boosts anxieties for climate change concerns, however, none of the studies has identified awareness of consequences’ silent role in identifying the anxieties associated with wasteful consumption practices—encapsulated in the concept of wastophobia. This study posits that it is more likely that heightened awareness of consequences can lead to the development of wastophobia in consumer behavior, characterized by feelings of fear, dismay, and shame. Thus, the present study postulates that heightened awareness of consequences can significantly activate wastophobia in consumer behavior.

*H_2_*: Awareness of consequences has a significant positive impact on wastophobia.

### 2.3 Wastophobia and consumer advocacy

The section of the literature review examines the association among wastophobia and various behavioral outcomes, including consumer advocacy, creative performance, moral courage, and pro-environmental conduct of consumers. Initially, *Consumer advocacy refers to activities that protect consumer interests, rights, promote informed choices, and encourage ethical practices. Advocacy for consumers’ aims to empower consumers’ concerns during the time of change* (Chelminski and Coulter, 2011 p. 362). Their study further indicates that consumer advocacy is related to complaining behavior; it empowers consumers to voice their dissatisfaction and adapt to change. Padhy (2015) explains that fear appeals significantly influence consumer advocacy in the context of smoking quitting behavior and brands acceptance (Jayasimha et al., 2017). However, no research has been conducted to date on the impact of wastophobia on consumer advocacy, particularly regarding the extension of product lifecycle by controlling planned obsolescence. In this context, we assume that potential customers would demand more sustainable and efficient appliances if their level of wastophobia grows. It is assumed that consumers who are more possessive towards e-waste and their consequences are more likely to seek products with extended lifespan. Consequently, this research proposes the following hypothesis in consideration of the arguments put forth by Jayasimha et al. (2017) and Parvatiyar and Sheth (2023). Therefore, based on the argument of Jayasimha et al. (2017) and Parvatiyar and Sheth (2023) this study postulates that wastophobia can significantly influence consumer advocacy towards sustainable electronic goods consumption and e-waste reduction.

*H_3a_*: Wastophobia has a significant positive impact on consumer advocacy.

### 2.4 Wastophobia and creative performance

*Creative performance is the ability to generate new and innovative ideas or solutions* to the complex problems (Amabile, 1993, p. 364). It encompasses numerous characteristics, including innovation, elegance, originality, and ability to solve poorly structured issues (Mumford and Gustafson, 2007). Intrinsic motivation is a powerful driver of creativity (Amabile, 1988) which unfolds over time in a systematic way (Clydesdale, 2006). Organizations aiming to improve their competitive position need to promote employees’ creative performance (Mumford et al., 2023). Moreover, Gabriel (2023) propagates that the fear of failure significantly enhances the creative performance of employees. Although the literature synthesizes that fear significantly impacts the creativity and creative performance of employees, there is a notable gap in the literature regarding the impact of wastophobia on consumer creative performance. Consumers with a high intensity of wastophobia will likely think more creatively, innovatively, and elegantly to develop innovative ideas for e-waste minimization by enhancing their creative performance. Therefore, building upon the argument of Mumford et al. (2023) and Gabriel (2023), the present study postulates that wastophobia can significantly enhance creative performance.

*H_3b_*: Wastophobia has a significant positive impact on creative performance.

### 2.5 Wastophobia and moral courage

*Moral courage is the tendency of individuals to act according to customs, norms, and values of society and remain steadfast in their views, even in the face of potential penalties, such as social exclusion* (Kidder and McLeod, 2005, p. 201). Past research has demonstrated that witnessing wrongdoing often inspires individuals’ moral courage (Miller, 2002) to perform ethically when they recognize the detrimental consequences of their choices (Aydin and Yildirim, 2021). Capoano et al. (2024) supported this claim by arguing that youngsters with a heightened awareness of the detrimental environmental consequences of their unsustainable consumption practices are more likely to adopt greener morals. Social cognitive theory supports this claim by arguing that moral courage is the outcome of personal, social, and environmental factors (Bandura, 2002). Thus, existing research synthesized that morally courageous individuals are more conscious of their actions (Ogunfowora et al., 2021) and their associated negative environmental costs (Hannah et al., 2011). Despite that, there remained a significant gap in the literature to explore the potential impact of wastophobia on moral courage. Despite that, there remained a significant gap in the literature to explore the potential impact of wastophobia on moral courage. Wastophobia may encourage moral courage, facilitating consumers to act ethically and align their behavior with ethical standards of sustainable consumption. Therefore, building on the insights from Kidder and McLeod (2005), Miller (2002), Bandura (2002), Ogunfowora et al. (2021), and Capoano et al. (2024), the present study hypothesizes that wastophobia can significantly promote moral courage.

*H_3c_*: Wastophobia has a significant positive impact on moral courage in consumer behavior.

### 2.6 Wastophobia and pro-environmental behavior

*Pro-environmental behavior encompasses actions aimed at minimizing environmental impact and promoting sustainability* (Schultz, 2014, p. 108). Past research portrays a positive association between climate change anxiety and environmentally responsible behavior (Clayton and Karazsia, 2020). Individuals who are more conscious of the detrimental effects of climate change are more likely to participate in environmentally responsible behaviors (Parvatiyar and Sheth, 2023). Furthermore, conscious consumer regarding climate change anxiety demonstrate their commitment to being good stewards of the environment and prefer greener behaviors (Shimul et al., 2024). However, there is a notable gap in the literature examining the role of wastophobia on pro-environmental behavior. None of the studies have examined how consumers respond to anxieties associated with wastophobia. To cover this gap, the present study posits that wastophobia can motivate consumers to rethink their consumption practices, especially when it comes to electronic consumption where there is a tendency to frequently discard electronic gadgets which are linked to environmental damage. Research has demonstrated that when consumers experience anxiety or fear-based emotions associated with wastophobia, they are more likely to adopt pro-environmental behavior. Therefore, based on the arguments of Clayton and Karazsia (2020), Parvatiyar and Sheth (2023), and Shimul et al. (2024), the presented study hypothesizes that consumers will respond more positively to develop pro-environmental behavior when they get familiar with wastophobia.

H_3d_ Wastophobia has a positive impact on pro-environmental behavior.

### 2.7 Mediation mechanism of wastophobia

According to González-Rodríguez et al. (2019), awareness challenges established norms and promotes *consumer advocacy* for a sustainable future. Awareness is the initial step that enables consumers to boost their *creative performance* as a means to align their actions with their values (Mumford et al., 2023; Brown and Treviño, 2014). Ganu (2018) highlighted that awareness of consequences often results in *moral courage*. In line with this, norm activation theory also postulates that awareness of consequences significantly activates personal norms and compels individuals to act morally (Schwartz, 1977). Parvatiyar and Sheth (2023) argued that awareness pushes individuals to strengthen their pro-environmental intentions for the preservation of the environment. The addressed literature synthesizes that awareness significantly promotes consumer advocacy, creative performance, moral courage, and pro-environmental behavior. However, none of the studies has attempted to examine the role of awareness in the context of recognizing one’s wasteful consumption practices and their consequential impact on discussed behavioral outcomes including consumer advocacy, creative performance, moral courage, and pro-environmental behavior through the mediation of wastophobia–fear of wasteful consumption. It is more likely that heightened wastophobia can significantly strengthen the association between awareness of wasteful consumption, awareness of consequences, consumer advocacy, creative performance, moral courage, and pro-environmental behavior. Therefore, based on the identified literature gaps, the present postulate that wastophobia can significantly mediate between awareness and multiple behavioral outcomes.

*H_4a_*: Wastophobia positively mediates between awareness of wasteful consumption and consumer advocacy.

*H_4b_*: Wastophobia positively mediates between awareness of wasteful consumption and creative performance.

*H_4c_*: Wastophobia positively mediates between awareness of wasteful consumption and moral courage.

*H_4d_*: Wastophobia positively mediates between awareness of wasteful consumption and pro-environmental behavior.

*H_5a_*: Wastophobia positively mediates between awareness of consequences and consumer advocacy.

*H_5b_*: Wastophobia mediates between awareness of consequences and creative performance.

*H_5c_*: Wastophobia mediates between awareness of consequences and moral courage.

*H_5d_*: Wastophobia mediates between awareness of consequences and pro-environmental behavior.

### 2.8 Theoretical foundations

**Figure 3:**
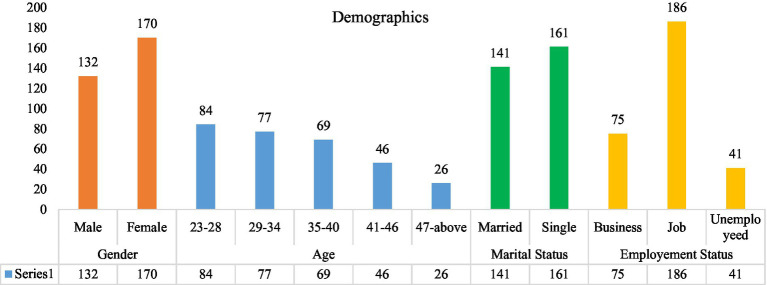
Demographical representation (Author generated).

This study employs two theoretical lenses including the theory of interpersonal behavior (TIB) (Donovan, 2011) and a comprehensive model of environmental psychology (Klockner, 2013) to develop a theoretical framework (see Figure 3). The key purpose is to explore the antecedents and consequences of wastophobia, which remained overlooked in the past literature. Additionally, to explore the association of cognitive and emotional factors to promote sustainable consumption behavior, and reduce the tendency of product discard, and planned obsolescence. TIB addresses the complexities in human behavior, which is difficult to predict accurately (Bravi et al., 2020). Past researchers highlight that solely focusing on cognitive aspects is inadequate for understanding the motivation behind wasteful consumption behavior and emphasizing the need for an integrated approach that combines cognitive and emotional factors (Filimonau et al., 2020). The TIB lens advocates this integration by postulating that behavioral responses toward e-waste are indeed complex and multifaceted (Ibrahim et al., 2018). Therefore, the study posits that cognitive (i.e., awareness of wasteful consumption and awareness of consequences) and emotional factors (i.e., wastophobia) would jointly promote and maintain sustainable consumption behavior. Therefore, the present research particularly investigates the contribution of cognitive factors (awareness of wasteful consumption and awareness of consequences), along with the emotional reactions associated with wastophobia (Hanif et al., 2022) to the reduction of e-waste. The major justifications for selecting these variables include: (a) contemporary studies highlight a significant relationship between emotions associated with waste (Roberts et al., 2017). In addition, (b) the improper disposal of waste causes emotional reactions such as guilt and grief, where guilt regarding the waste can motivate individuals to reassess their behavior on moral grounds (Clayton and Karazsia, 2020). Finally, (c) it is also true that moral courage is related to having self-determination (Karbasi, 2024) because it can encourage responsible consumption practices and discourage practices that negatively affect the environment for a long time.

The CMEP model simultaneously grasps the core psychological aspects that motivate individuals to act in favor of maintaining a healthy environment (Klockner, 2013). Central to this model, wastophobia is incorporated as a significant emotional factor, which encompasses feelings of fear, shame, guilt, and dismay. The study posits that when consumers experience heightened wastophobia, they become more likely to be aware of the consequences that are posed by inefficient consumption practices, triggering feelings of guilt, that lead to behavioral changes and planning for waste accumulation. This framework postulates that fostering emotional engagement through boosting wastophobia enhances consumer advocacy, moral courage, creative performance, and pro-environmental behaviors, consequently promoting sustainable consumption practices by addressing the challenges of e-waste.

### 2.9 Research model

The combination of TIB and CMEP offers a robust integrated theoretical framework (see Figure 2) for comprehensively understanding the mechanism of cognitive and emotional factors influencing e-waste reduction intentions. The presented model overcomes the shortcomings of behavioral theories, such as the theory of planned behavior, which does not adequately explain the role of emotions, its weaker explanatory power, and the complexities associated with behavioral intentions. Specifically, this model provides a ground to understand the factors that can contribute to the occurrence of wastophobia as well as the repercussions of the phenomena on sustainable consumption behavior. In this context, the variables including awareness of wasteful consumption, awareness of consequences, consumer advocacy, moral courage, and creative performance are taken from TIP. While emotional factors including pro-environmental behaviors and wastophobia emanate from CMEP. The approach not only boosts the explanatory power of the integrated model but also provides guidelines to develop innovative targeted behavioral strategies to foster sustainable consumption behavior. The definitions of the study measures are presented in Table 1.

**Table 1:** Definition of measures.

Measures	Variables	Definition	References
Awareness of wasteful consumption	Independent	Awareness of wasteful consumption refers to the understanding and recognition of the excessive or inefficient use of resources	Hanif et al. (2021)
Awareness of consequences		A situation in which an individual understands the consequences of their actions, such as the harm to people, communities, and ecosystems	Ryan and Spash (2012)
Wastophobia	Meditator	A state of fear that promotes the considerate behavior and deters the way individual develops mental precociousness, apprehension, and the practices of wasteful consumption. It may give rise to dismay and feel an individual culprit, decrepit and accountable for wasteful consumption practices which are inconsiderate”	Hanif et al. (2022)
Consumer advocacy	Dependent	Consumer activities which protect consumer interests and rights, informing choices as well as encouraging ethical practices.	Jayasimha et al. (2017)
Creative performance		Consumers’ ability to generate new and innovative ideas or solutions.	Amabile (1993)
Moral courage		Moral courage is the tendency of individuals to confront ecological hazards and remain steadfast in their views, even in the face of potential penalties, such as social exclusion.	Kidder and McLeod (2005)
Pro-environmental behavior		Pro-environmental behavior encompasses actions aimed at minimizing environmental impact and promoting sustainability.	Schultz (2014)

## 3 Methodology

A quantitative research survey approach was designed to explore the antecedents and consequences of wastophobia. This section provides a comprehensive overview of the research context, population and sampling, measurement scale, and data collection process and analysis technique.

### 3.1 Research context

China produces 68 million metric tons of electronic waste, representing 20% of the worldwide e-waste (Wang et al., 2024). Domestic consumers are the largest contributor to e-waste as well as enormous (90%) CO_2_ emissions of the overall electronic industry (Zhang et al., 2024). Hence, understanding the role of Chinese households in this context could help to devise strategies to reduce electronic waste.

### 3.2 Population, sampling unit, sampling technique, and sample size

Data was collected from electronics goods consumers living in Shenzhen city, China, as discussed earlier China is the leading economy in electronic goods production, consumption, waste generation, and CO_2_ emission (Zhang et al., 2024), while Shenzhen is renowned for electronics goods manufacturing and consumption (Dong et al., 2024). The study identified the sampling unit as the consumers of electronic gadgets. The non-probability convenience sampling technique was followed to obtain a representative sample of the researched population, thus aiding in the collection of primary data that depicts different views and experiences of consumers. By doing so, it seeks to enhance the reliability and validity of results, thus deepening comprehension of wastophobia. For the sample size selection, two criteria were considered (a) a minimum of five responses per item estimate and (b) a sample size of 300 or more for structural equation modeling. The total measurement items were 25 in the current study. Considering the rule of five cases per parameter estimate, 125 responses were sufficient for examining the research hypotheses. However, as encouraged in the literature, it is desirable to use a large sampling to minimize the effects of sampling error (Wolf et al., 2013).

### 3.3 Measurement scale

This study considered a 5-point Likert scale research questionnaire (5 represents “strongly disagree” and 1 represents “strongly agree”) for data collection purposes. The constructs in the questionnaire included awareness of wasteful consumption (Dewaters et al., 2013), awareness of consequences (Ryan and Spash, 2012) wastophobia (Marks and Mathews, 1979), consumer advocacy (Jayasimha et al., 2017), creative performance (Meinel et al., 2018), moral courage (Hannah et al., 2011), and pro-environmental behavior (Parvatiyar and Sheth, 2023) was adopted from the prior studies (see Table 1). To minimize the language barrier, the adopted scale was translated from English into Chinese and back-translated into English to ensure the equivalence in the meaning of the items in the scale (Brislin, 1986).

### 3.4 Data collection process

Data were collected in multi-waves (i.e., Time frame 1 and Time frame 2) to mitigate common method bias issue as recommended by Podsakoff et al. (2003). Initially, we visited 20 mega electronic outlets and obtained consent from 13 retailers to facilitate data collection from their actual customers. We set up 10 stalls outside the identified retail outlets and engaged only with those customers who actually purchase new electronic gadgets and recently discarded old gadgets. Each customer received a brief introduction to the study to obtain their informed consent. During time frame 1 (April 2024—May 2024), we gathered 410 responses related to constructs including awareness of wasteful consumption, awareness of consequences, and wastophobia. In the time frame 2 (August 2024—September 2024), a questionnaire containing constructs including consumer advocacy, creative performance, moral courage, and pro-environmental behavior was distributed among the same (410) consumers after a month time break (through online channels including WeChat and emails) and got back 330 questionnaires. The two-month (June–July) time-lag process facilitates ensuring the generalizability of the findings (Austin and Stuart, 2015).

A demographic assessment of the obtained responses (*n* = 302) revealed that 170 (56%) were female participants, 84 (28%) were in the 23- to 28-year-old age range, 141 (47%) were married, and 186 (62%) were doing jobs. The demographics of our sample demonstrate that the sample of the study was mature enough to comprehend the language and terminology utilized in the survey instruments.

## 4 Data analysis and results

Data analysis was performed in SPSS and AMOS-24, which facilitates simultaneous estimations of measurement and structural models. To ensure data accuracy, data cleansing was performed on 330 returned questionnaires, addressing missing values, outliers, and normality before model testing (Ganti and Sarma, 2022). Following Sekaran (2006) recommendations, 22 questionnaires with more than 10% of missing data were removed. Outlier analysis using the Mahalanobis distance method (*p* < 0.001) has led to the exclusion of 6 cases (Kline, 2014). Additionally, normality tests were conducted on the remaining sample size of 302 participants. The results presented in Table 2 portrays skewness (−1.065–0.678.) and Kourtosis (−0.554 to 1.437) range within the acceptable threshold established by Mishra et al. (2019) (Skewness ± 2, and Kourtosis ± 4), as well as threshold proposed by Kline (2011) (Skewness ± 3, and Kourtosis ± 10); and Hair et al. (2010) (skewness ±2, and Kourtosis ± 7) respectively for sample exceeding 300 participants. Additionally, Shapiro–Wilk test and Kolmogorov–Smirnov were also conducted to examine the data normality. The extracted statistical findings range for Shapiro–Wilk test (0.123–0.241, *p* > 0.05) and Kolmogorov–Smirnov test (0.101–0.261, *p* > 0.05), remained higher than the addressed benchmark values (*p* > 0.05) for variables AWC, AC, WP, CL, CP, MC and PEB (see Table 2), as addressed by Steinskog et al. (2007). The adherence of skewness and Kurtosis, Kolmogorov–Smirnov, and Shapiro–Wilk test has ensured the normality of the dataset, thereby facilitating the appropriateness of subsequent analysis that relies on normality assumptions. Harman’s single-factor test was used to calculate the potential issue of common method bias (CMB) in the dataset. The results revealed that a single dominant factor accounted for only 38.92% of the variance, which is lower than the cutoff value of 50%, below the cutoff recommended by Podsakoff et al. (2003). The results conclude that CMB is not a potential concern in the available dataset. Finally, the confirmatory factor analysis (CFA) technique was confirmed for data analysis purposes as the data meets the sample size and multivariate requirements of normality (Mishra et al., 2019).

**Table 2:** Normality analysis.

Measures	Skewness	Kourtosis	Shapiro–Wilk			Kolmogorov-Smirnov^a^	
			df	Statistic	Sig.	Statistic	Sig.
AWC	0.104	−0.170	302	0.212	0.119	0.191	0.112
AC	−1.065	1.014	302	0.241	0.141	0.261	0.164
WP	0.490	0.156	302	0.123	0.083	0.112	0.079
CL	−0.255	−0.554	302	0.170	0.139	0.101	0.056
CP	0.447	0.206	302	0.186	0.140	0.149	0.063
MC	0.678	1.437	302	0.235	0.140	0.189	0.121
PEB	0.371	−0.197	302	0.151	0.121	0.118	0.073

a Lilliefors significance correction.

### 4.1 Discriminate validity, cross loadings

Discriminant validity assessed the uniqueness of the construct, was assessed using the cross-loadings proposed by Hair et al. (2013). Discriminant validity is established when the items demonstrate higher loadings on their intended constructions relative to cross-loadings, as highlighted in bold in Table 3. Additionally, the results depicted that all the constructs exhibited significant loadings > 0.40 (Hair et al., 2013), and also fulfilled uni-dimensionality requirements (Mishra et al., 2019). This study established discriminant validity since all the cross-loadings of items were lower by 0.20 (Hair et al., 2010) when compared inside the same construct.

**Table 3:** Discriminant validity—cross loadings.

Items	AC	CA	CP	PEB	AWC	MC	WP
Environmental protection will provide a better world for me and my children.	**0.811**	−0.11	0.056	−0.06	0.069	0.093	0.005
Protecting the environment will threaten jobs for people like me.	**0.758**	−0.05	0.045	0.12	−0.09	0.06	0.046
Environmental protection will help people have a better quality of life	**0.755**	0.041	0.069	0.007	0.015	−0.12	0.091
The effects of pollution on public health are worse than we realize.	**0.753**	−0.00	−0.02	0.045	0.009	0.047	0.074
Over the next several decades, thousands of species will become extinct.	**0.748**	−0.01	0.042	−0.02	0.162	−0.09	0.041
Saving energy is important	0.023	**0.79**	−0.03	−0.09	0.092	0.059	−0.01
I am willing to buy fewer things to save energy	0.087	**0.77**	0.047	−0.03	−0.1	0.082	−0.1
Many of my everyday decisions are affected by my thoughts on energy use	−0.052	**0.70**	−0.04	−0.07	−0.15	−0.11	0.028
Feeling miserable or depressed	−0.172	**0.54**	−0.04	0.114	0.013	−0.11	0.077
Feeling irritable or angry	0.025	0.017	**0.79**	0.143	0.021	0.067	0.088
Feeling tense or panicky	0.062	−0.04	**0.75**	0.059	0.058	0.057	0.146
It makes me feel good when I tell others about the bad experiences with the waste decision I took.	0.076	−0.04	**0.72**	0.091	0.106	0.215	0.112
I feel relieved after sharing with others my bad experience with products or services	−0.069	−0.05	0.019	**0.74**	−0.05	0.186	0.148
I often warn others about bad goods hoping that they will share similar information with me.	0.095	−0.10	0.02	**0.67**	0.167	0.292	−0.1
If I warn others, they will warn me about the bad product/service	0.032	0.167	0.083	**0.64**	0.099	0.011	0.124
Performance accomplishments aim at building participants’ creative self-belief	0.056	−0.14	0.29	**0.62**	−0.07	0.115	0.04
Experience incorporates participants’ observations of the classmates being creative	−0.004	−0.01	0.089	0.006	**0.76**	0.122	0.058
Verbal persuasion aims at convincing participants verbally that they are capable and creative.	0.078	−0.10	0.078	0.181	**0.74**	−0.14	0.026
Courage to overcome perceived threat to do what is right, even when faced with peer pressures.	0.066	−0.03	0.006	−0.06	**0.68**	0.184	0.217
Courage to correct others who behave inappropriately	−0.121	−0.00	0.215	0.15	0.005	**0.69**	0.046
Demonstrates courage to do the right thing, even at personal cost.	0.066	−0.11	−0.03	0.218	0.026	**0.69**	−0.02
I would help raise money to protect nature.	0.026	0.051	0.24	0.132	0.171	**0.66**	0.184
I try to tell others that nature is important.	0.091	0.017	0.149	0.114	0.049	−0.16	**0.77**
Plants and animals have as much right as humans to exist.	0.104	0.025	0.011	0.044	0.155	0.146	**0.71**
I always turn off the light when I do not need it anymore.	0.052	−0.06	0.281	0.07	0.086	0.193	**0.67**

Extraction: Principal Component Analysis. Rotation: Varimax (Kaiser Normalization). Bold values shows factor structure.

### 4.2 Reliability, validity, and correlations

The internal consistency of items was measured through composite reliability, known as construct reliability. Hair et al. (2010) proposed the threshold value (> = 0.70) of composite reliability (CR). The extracted CR values (0.88–0.71) were deemed reliable for subsequent analyses. Moreover, the convergent validity determines whether the constructs converge or diverge. Convergent validity was accomplished as the average variance extracted (AVE) value (0.67–0.77) exceeded the threshold of 0.50 (Hair et al., 2013). When it comes to determining the reliability of a scale, Cronbach’s alpha (*α*) is most suitable. The minimum threshold for Cronbach’s Alpha is > 0.60 (Kline, 2014). The reliability of all scales is ensured as all alpha values remained greater than 0.60. The mean values represent the variable that is practiced more comparatively. The high mean score of awareness of consequences (3.93) indicated a high level of awareness of consequences, whereas the low mean score of PEB (1.83) indicated a low level of PEB among consumers. Nevertheless, the data for CA have high standard deviations, specifically 0.92, in comparison to other variables. The Pearson correlation analysis method was carried out to examine the association between each pair of variables examined (Armstrong, 2019). Based on the extracted correlation findings, awareness of wasteful consumption (AWC) and awareness of consequences (AC) indicated a substantial positive association with wastophobia (*r* = 0.279**; *r* = 0.175***) respectively. Furthermore, it is worth noting that wastophobia has a noteworthy positive connection with creative performance (*r* = 0.354**), moral courage (*r* = 0.290, *ρ* < 0.01), and pro-environmental behavior (*r* = 0.274**), except consumer advocacy (*r* = −0.010). These findings presented in Table 4 are theoretically justified (Hair et al., 2013).

**Table 4:** Reliability, validity, descriptive, and correlation analyses.

Measures	C.R.	AVE	*a*	Means	SD	AWC	AC	WP	CA	CP	MC	PEB
AWC	0.77	0.73	0.6	2.06	0.6	1						
AC	0.88	0.77	0.8	3.93	0.8	0.125^*^	1					
WP	0.71	0.72	0.6	1.95	0.6	0.279^**^	0.175^**^	1				
CA	0.80	0.71	0.7	2.97	0.9	−0.143^*^	−0.07	−0.1	1			
CP	0.80	0.76	0.7	1.99	0.7	0.185^**^	0.134^*^	0.354^**^	−0.1	1		
MC	0.72	0.68	0.6	2.01	0.6	0.256^**^	0.09	0.290^**^	−0.1	0.556^**^	1	
PEB	0.76	0.67	0.7	1.83	0.6	0.183^**^	0.07	0.274^**^	−0	0.414^**^	0.467^**^	1

AWC, Awareness of Wasteful Consumption; AC, Awareness of Consequences; WP, Wastophobia; CA, Consumer Advocacy; CP, Creative Performance; MC, Moral Courage; PEB, Pro-environmental Behavior.

### 4.3 Measurement model fit

**Figure 4:**
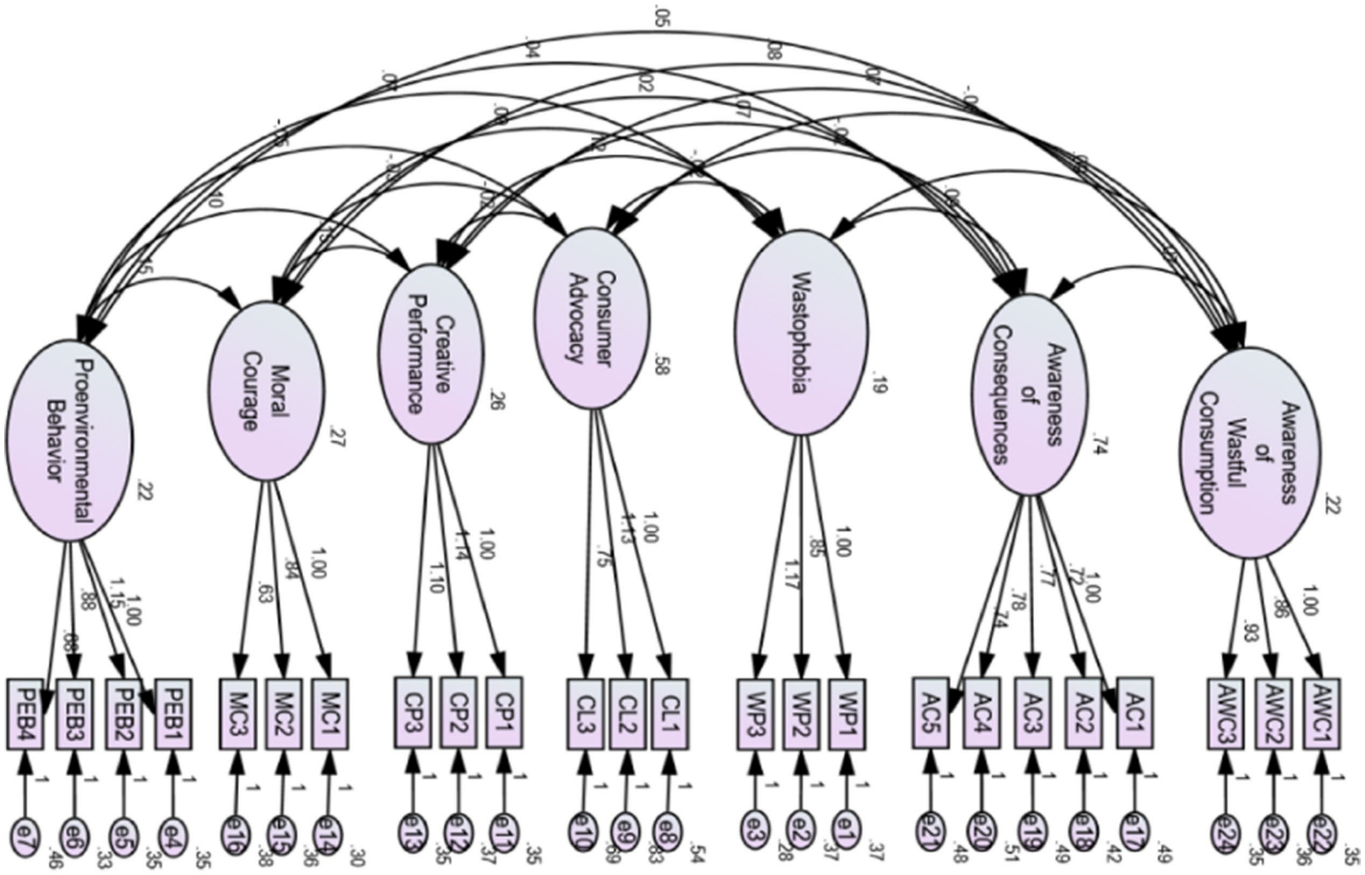
Factor modeling.

The model fit was assessed in the measurement model testing phase of confirmatory factor analysis using various fit indices including the degree of freedom index (*χ* 2 / DF < 3), Tucker–Lewis index (TLI ≥ 0.90), the incremental-fit index (IFI ≥ 0.90), comparative-fit index (CFI ≥ 0.90), goodness of fit index (GFI ≥ 0.90), the standardized root mean square residual (RMSR ≤ 50), and root mean square error of approximation (RMSEA≤ 50) (Awang, 2012). The analysis initiated with a single factor model 1 and progressed through multiple iterations, examining two-factor model, three-factor model, four-factor model, five-factor model, and six-factor model, consequently culminating in seven-factor model (see Table 5). The fit indices (GFI, IFI, TLI, CFI, SRMR, RMSEA) for factor model 1 to 5 remained lower than the identified benchmark values, indicating unsatisfactory model fit. The factor model 6 showed slight improvements, but it still fell short of the benchmark criteria. The final factor model 7 demonstrating excellent fit, with values for χ 2 / DF 1.388, GFI, 0.916, IFI = 0.939, TLI = 0.926, CFI = 0.938, SRMR = 0.036, and RMSEA = 0.036, respectively. This model effectively balances the simplicity and complexity, thereby ensuring the reliability and generalizability of the model. Notably, during the modeling process, autocorrelation (error terms correlation) through modification indices (MIs) was assessed. The findings of model 7 revealed no association among error terms in the benchmark model 7 (see Figure 4) while other model fit indices (discussed above) has achieved the benchmark criterion, as suggested by Brown (2015), which illustrates not to remove any item from the final model. The absence of necessary adjustment in modification indices ensured that the model structure was data driven and theoretically sound (Byrne, 2016), prioritizing the integrity of construct over statistical alterations.

**Table 5:** Fit statistics from measurement model comparison.

Models	χ^2^	DF	χ^2^/DF	GFI	IFI	TLI	CFI	SRMR	RMSEA
Factor Model 7	352.693	254	1.388555	0.916	0.939	0.926	0.935	0.035	0.036
Factor Model 6	478.605	260	1.840788	0.882	0.865	0.841	0.862	0.046	0.053
Factor Model 5	628.509	265	2.371732	0.837	0.775	0.74	0.77	0.068	0.068
Factor Model 4	673.987	269	2.505528	0.837	0.749	0.715	0.744	0.068	0.071
Factor Model 3	740.694	272	2.723140	0.822	0.709	0.673	0.673	0.704	0.076
Factor Model 2	817.008	274	2.981781	0.807	0.663	0.624	0.657	0.073	0.081
Factor Model 1	1274.672	275	4.635171	0.697	0.378	0.311	0.368	0.103	0.110

χ^2^, Chi-square; DF, Degree of freedom; GFI, Goodness of fit index; IFI, Incremental fit index; TLI, Tucker–Lewis index; CFI, Comparative-fit index; SRMR, standardized root mean square residual; RMSEA, root mean square error of approximation.

### 4.4 Hypothesis testing

**Figure 5:**
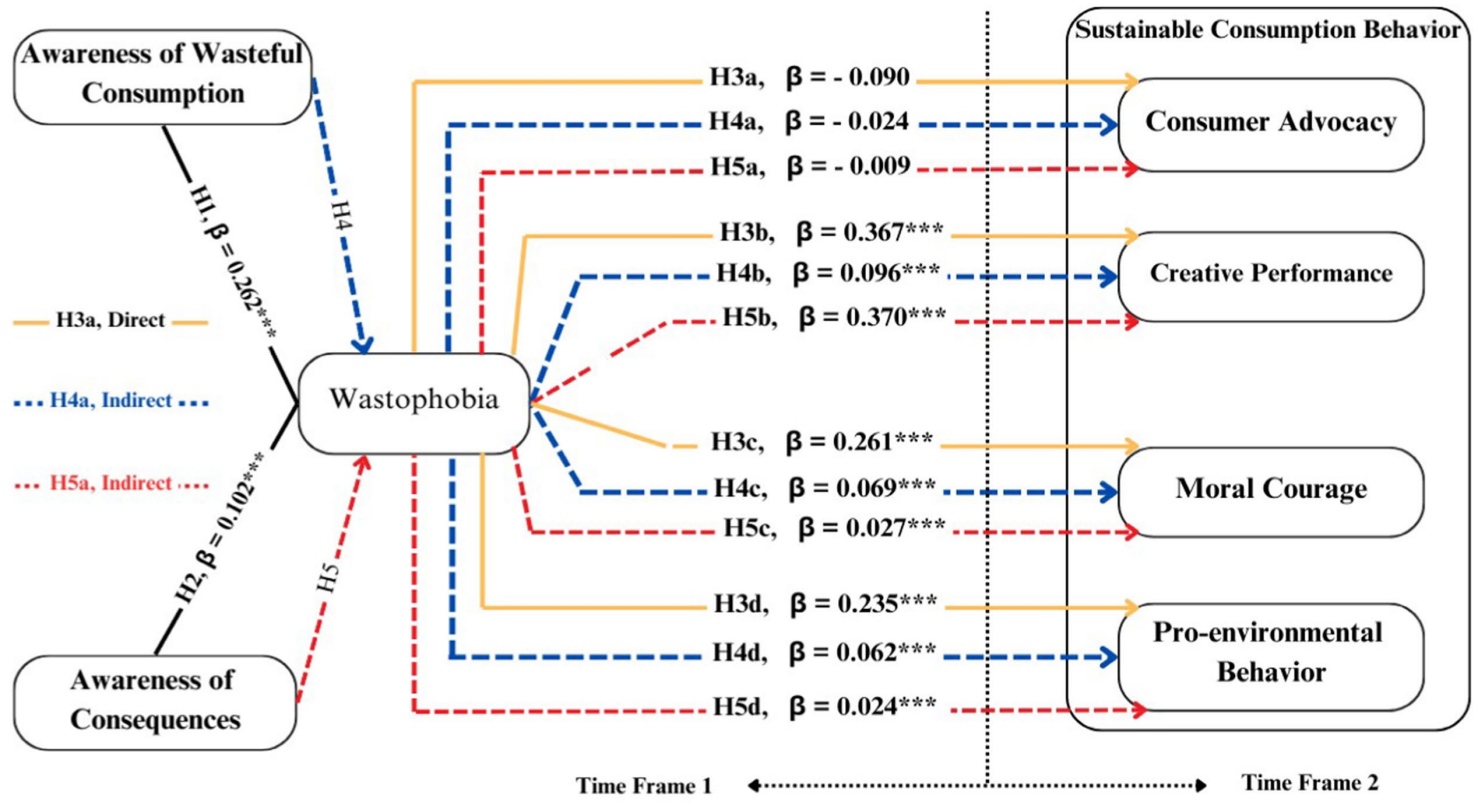
Measurement model.

The results of the measurement model indicated a significant impact of AWC (H_1_: *β* = 0.262, *p* < 0.001) and AC (H_2_: *β* = 0.102, *p* < 0.001) on wastophobia. While, the impact of wastophobia on creative performance (H_3b_: *β* = 0.367, *p* < 0.001), moral courage (H_3c_: *β* = 0. 0.261, *p* < 0.001), and pro-environmental behavior (H_3d_: *β* = 0.235, *p* < 0.001) remained statistically significant at 1% confidence interval. However, the wastophobia indicated a negatively insignificant impact on consumer advocacy (H_3a_: *β* = −0.090, *p* > 0.01) (see Direct effects in Table 6 and Figure 5).

**Table 6:** Path analysis (standardized weights).

	Independent	Dependent	Estimate	S.E.	C.R.	*p*	Decision
H_1_	Awareness of Wasteful Cons ➜	Wastophobia	0.262	0.055	4.733	***	Accepted
H_2_	Awareness of Consequences ➜	Wastophobia	0.102	0.039	2.573	***	Accepted
H_3a_	Wastophobia ➜	Consumer advocacy	−0.090	0.099	−0.909	0.363	Rejected
H_3b_	Wastophobia ➜	Creative performance	0.367	0.065	5.627	***	Accepted
H_3c_	Wastophobia ➜	Moral courage	0.261	0.064	4.069	***	Accepted
H_3d_	Wastophobia ➜	Pro-environmental behavior	0.235	0.057	4.12	***	Accepted

***(*p* < 1%), **(*p* < 5%).

The mediating relationships were assessed using the bootstrap method (N = 10,000 at 95% confidence interval). The mediating mechanism of wastophobia (WP) among awareness of wasteful consumption and consumer advocacy (AWC ➜ WP ➜ CA), creative performance (AWC ➜ WP ➜ CP), moral courage (AWC ➜ WP ➜ MC), and pro-environmental behavior (AWC ➜ WP ➜ PEB) was examined through direct, indirect effects, their level of significance, and their lower and upper boundaries. The same criteria were followed to assess the mediation mechanism of Wastophobia for awareness of consequences.

The findings presented in Table 7 represented the mediation mechanism of wastophobia. The direct effect of AWC on CA was negative and significant (*β* = −0.122, LL = −0.356, UP = −0.038, *p* < 0.05), while the indirect effect remained negative but insignificant. Moreover, upper and lower limits are summed-up at zero (*β* = −0.024, LL = −0.078, UP = 0.022, *p* > 0.10). The findings concluded that wastophobia has no significant mediating impact on consumer advocacy. Thus, the study rejects the hypothesis. H4a. In hypothesis H_4b,_ AWC effect was empirically tested on CP considering the mediating effect of wastophobia. The results indicated that the direct effect of AWC remained positive but insignificant. The upper and lower limits were found to be zero and probability values remained insignificant at 10% (*β* = −0.024, LL = −0.008, UP = 0.022, *p* > 0.10). On the other side, the indirect effect was found significant at 1% (*β* = 0.096, LL = 0.048, UP = 0.155, *p* < 0.01). These results concluded that wastophobia fully mediates the relationship between AWC and CP. Thus, the study accepted hypothesis H_4b._

**Table 7:** Mediation analysis using bootstrap.

Relationships	Estimate	*p*-values	Bootstraps at 95%		Hypotheses	Decision
			LL	UL		
H4a. AWC ➜ WP ➜ CA					No Mediation	Rejected
Direct effect	−0.122	**	−0.356	−0.038		
Indirect effect	−0.024	0.405	−0.078	0.022		
H4b. AWC ➜ WP ➜ CP					Full Mediation	Accepted
Direct effect	0.088	0.125	−0.008	0.21		
Indirect effect	0.096	***	0.048	0.155		
H4c. AWC ➜ WP ➜ MC					Partial Mediation	Accepted
Direct effect	0.187	***	0.097	0.327		
Indirect effect	0.069	***	0.029	0.116		
H4d. AWC ➜ WP ➜ BEP					Partial Mediation	Accepted
Direct effect	0.114	**	0.013	0.213		
Indirect effect	0.062	***	0.025	0.104		
H5a. AC ➜ WP ➜ CA					No Mediation	Accepted
Direct effect	−0.059	0.394	−0.184	0.054		
Indirect effect	−0.009	0.406	−0.033	0.008		
H5b. AC ➜ WP ➜ CP					Full Mediation	Accepted
Direct effect	0.056	0.194	−0.016	0.129		
Indirect effect	0.370	***	0.014	0.065		
H5c. AC ➜ WP ➜ MC					Full Mediation	Accepted
Direct effect	0.025	0.61	−0.058	0.106		
Indirect effect	0.027	***	0.008	0.049		
H5d. AC ➜ WP ➜ BEP					Full Mediation	Accepted
Direct effect	0.013	0.739	−0.051	0.075		
Indirect effect	0.024	***	0.007	0.044		

Bootstrapping sample, *N* = 5,000.

The mediating effect of wastophobia between AWC and MC was empirically tested in Hypothesis H_4c_. The statistical results portrayed that both the direct (*β* = 0.187, LL = 0.097, UP = 0.327, *p* < 0.01) and indirect effects (*β* = 0.069, LL = 0.029, UP = 0.116, *p* < 0.01) remained statistically significant. While no zero value was observed in the upper and lower boundaries. Thus, the study concludes that wastophobia partially mediates the relationship between AWC and MC. Hence, hypothesis H_4c_ is accepted. In hypothesis H_4d,_ the mediating effect of wastophobia was empirically tested between AWC and BEP. The findings depicted that AWC has significant positive direct effects on PEB (*β* = 0.114, LL = 0.013, UP = 0.213, *p* < 0.05). While the indirect effect was found significantly positive (*β* = 0.062, LL = 0.025, UP = 0.104, *p* < 0.01). The findings concluded that wastophobia partially mediates between AWC and BEP. Thus, the study accepted hypothesis H_4d_.

The role of AC on CA in the presence of Wastophobia was examined in hypothesis H_5a_. The results depicted that the direct effect of AC on CA remained statistically negative but insignificant (*β* = −0.059, LL = −0.184, UP = 0.054, *p* > 0.10). Moreover, the indirect mediating effect of wastophobia between AC on CA also remained negative but insignificant (*β* = −0.009, LL = −0.033, UP = 0.008, *p* > 0.10). We found zero in the upper and lower limits of direct and indirect effects. Based on these findings, the study concluded that wastophobia has no mediation effect and hypothesis H_5a_ is rejected. Further, the mediating role of wastophobia between AC and CP is empirically assessed in hypothesis H_5b._ The result portrayed that the direct effect of AC on CP was found positive and insignificant (*β* = 0.056, LL = −0.016, UP = 0.129, *p* > 0.10). While the indirect effect was found statistically significant positive (*β* = 0.37, LL = 0.014, UP = 0.065, *p* < 0.01). The study concludes that wastophobia fully mediates the relationship between AC and CP and thus hypothesis H_5b_ is accepted.

In hypothesis H_5c,_ the mediating role of wastophobia was empirically assessed between AC and MC. The results indicated that the direct effect of AC on MC was found statistically positive but insignificant (*β* = 0.025, LL = −0.058, UP = 0.106, *p* > 0.10). We found zero in the upper and lower limits during the bootstrapping process. While the indirect mediating effect of wastophobia remained statistically significantly positive between AC and MC (*β* = 0.027, LL = 0.008, UP 0.049, *p* < 0.01). The findings concluded that wastophobia fully mediates the relationship between AC and MC. The study accepted hypothesis H_5c_. Finally, the mediating role of wastophobia was examined between AC and PEB. The results indicate that the direct effect of AC and PEB was found statistically positive but insignificant (*β* = 0.013, LL = −0.051, UP 0.075, *p* > 0.01). Moreover, the mediating indirect effect of wastophobia was found statistically significant and positive (*β* = 0.024, LL = 0.007, UP 0.044, *p* < 0.01). The results portrayed that wastophobia significantly strengthens the association between AC and PEB. Thus, the study accepted the hypothesis H_5d_.

## 5 Discussion

The study found a significant association between awareness of wasteful consumption, awareness of consequences, and wastophobia, validating hypotheses H_1_ and H_2_. The findings support the altruism behavioral philosophy claiming that heightened awareness motivates consumers to recognize self-determination (Klockner, 2013). Moreover, consumers felt guiltier and were more aware of their actions’ contribution to e-waste generation, and their consequential impacts on the environment. These findings also support the idea presented in the comprehensive model of environmental psychology and climate change anxiety which profoundly addresses that emotional responses get activated as consumers become aware of environmental implications (Clayton and Karazsia, 2020; Graham-Rowe et al., 2015). Thus, this study concluded that awareness of wasteful consumption and awareness of consequences significantly activate fear, guilt, and shame of wasteful consumption practices in consumer behavior. Therefore, the study claims that awareness of wasteful consumption and awareness of consequences are the fundamental antecedents of wastophobia.

The study suggests that wastophobia makes consumers uneasy because waste anxieties might inspire constructive and innovative thinking and action. This is supported by the claim presented in the study of Burch and Widman (2021), which suggests that negative emotions can inspire innovative environmental solutions. This is an important consideration because it means that wastophobia is a source of creative energy that can be channeled for use by environmental campaigns to encourage consumers to be more creative about reducing e-waste, thus validating hypothesis H_3b_. Moreover, the research results about moral courage highlight the fact that emotions can indeed facilitate ethical decision-making. If consumers within the wastophobia frame of mind are confronted with sentiments of disgust concerning \wasted\ resources, they will be motivated to take a stand against such wasteful behaviors – a notion supported by Graham-Rowe et al. (2015) who asserted that moral emotions help propel a person to behave in line with their principles. This connection underlines the need to cultivate a wastophobia culture that might support moral courage to encourage a more stringent adherence to sustainable consumption practices among the consumers, thus validating H_3c_.

Moreover, the observed relationship between wastophobia and pro-environmental behavior is consistent with earlier work, which demonstrates that fear can enhance the willingness of people to buy green behaviors (Kollmuss and Agyeman, 2002). Wastophobia consumers may engage in extending product lifecycle or buy green products because they feel a strong concern towards protecting the environment. This goes back to the assertion that people undertake pro-environmental behavior which involves emotional engagement, and this suggests that those who communicate about sustainability to the general public, especially politicians and campaigners, should include emotions in their appeal if they want to achieve maximum public commitment to the practice, thus the study validated hypothesis H_3d_.

Nevertheless, wastophobia’s weak association with consumer advocacy raises some issues that require further examination in subsequent studies. Fear is understandable if it leads to actions at the individual and the group levels. However, mobilizing people for advocacy may require higher-order motivational processes and mechanisms. This implies that there may be an incomplete understanding of the literature on the factors determining the willingness or unwillingness of consumers to engage in advocacy behavior about emotions experienced. More attention needs to be paid to these aspects in future studies with a focus on how to create cohesion and a sense of purpose among those who hold wastophobia in a bid to get them to policy advocate for structural redevelopment, thus rejecting H_3a_.

The mediating interactions revealed the significant positive effect of wastophobia on multiple behavioral outcomes. Wastophobia fully mediated the connection between AWC-creative performance, AC-creative performance, AC-moral courage, and AC-Pro-environmental behavior, with strong indirect effects operationalizing hypotheses H_4b,_ H_5b,_ H_5c,_ and H_5d_. However, wastophobia partially mediated the AWC-moral courage (MC) and AWC-pro-environmental behavior (PEB) connections, validating hypotheses H4c and H4d. Consumer advocacy (CA) and wastophobia had negative direct correlations and negligible mediation in AWC and AC settings, hence hypotheses H_4a_ and H_5a_ were rejected. These findings suggest that wastophobia serves as a catalyst to promote consumer creative performance, moral courage, and pro-environmental behavior but not consumer advocacy. The findings emphasize the relevance of emotion in consumer behavior and offer novel ways to promote wastophobia as a motivator of sustainable consumption behavior. The study also implies that consumers may join in advocacy under undefined situations, which needs to be explored in future studies.

### 5.1 Theoretical contributions

This research enriches the theoretical literature in several key dimensions including consumer psychology, sustainable production and consumption, and environmental psychology. First, this study identified two fundamental antecedents of wastophobia including awareness of wasteful consumption and awareness of consequences. The study suggested that these antecedents (AWC, AC) are interrelated but played distinct role in activating wastophobia in consumer behavior. Importantly, consumers are more fascinated towards information seeking for their wasteful consumption practices as they perceive that such behavior is directly concerning to their routine actions raising personal expenses. Likewise, the AC proved a driving force that increases the degree of danger perceived from waste or inefficient consumption activities on the environment and individual well-being. These findings are linked to the past study of Hanif et al. (2022) which highlighted the significant role of AWC on wastophobia. Our study advances the discourse by suggesting that not only AWC but also AC plays a crucial role, thereby offering a more comprehensive understanding of the psychological drivers behind wastophobia.

Second, this research made an important contribution by exploring the consequences of wastophobia. The findings suggest that heightened wastophobia triggers a chain reaction and alleviates multiple consequences including CP, MC, and PEB. Wastophobia enforces consumers to think critically, perform creatively, morally, and take meaningful environmentally friendly steps to minimize their inefficient resource consumption practices, oppose disposability culture, and promote a sustainable future. This contribution reinforces the Conservation of Resources (COR) theory, which states that changes in behavior arise from perceived threats against valued resources (Arkorful et al., 2023). In addition, the study found that wastophobia served as an intervening variable between AWC, AC, and the identified behavioral outcome. Although the direct path remained significant but the presence of wastophobia as a mediating factor, improved the explanatory powers of CP, MC, and PEB.

The introduced groundbreaking theoretical research framework centered on the wastophobia construct contributes to the literature of TIB and CMEP. Wastophobia performed as a mediating mechanism and full mediation was observed among awareness of consequences, moral courage, and pro-environmental behavior. While, fully mediates between awareness of wasteful consumption, awareness of consequences, moral courage, creativity, and pro-environmental behavior. The findings revealed that the direct effect is lower than the indirect effect. It indicated that emotions make it possible for people to follow rules that promote pro-social behavior driven by cognizance of things happening around them. Therefore, this evidence reinforces NAT’s proposition that cognitive understanding and affective responses act as triggers to moral norms hence providing a deeper comprehension of how knowledge transforms into action.

### 5.2 Practical implications

The findings of this research are well aligned with the United Nations Sustainable Development Goals—embedded within the UN 2030 agenda and China Dual Carbon Neutrality Goals 2030–2060 which is an action plan for sustainable development focusing on issues like climate change (Pal et al., 2021). The conclusions drawn from the study have therefore far-reaching implications for various consumer, governments, organizations, and societal perspectives.

Initially, the study suggests policymakers to utilize awareness of wasteful consumption and awareness of consequences in their promotional strategies to educate consumer about their electronic waste practices and their environmental implications. Heightened awareness of wasteful consumption practices should enable consumers to comprehensively understand how their conscious and unconscious inefficient consumption practices reduce the lifecycle of usable products. It should also inform the consumers about the destructive consequences of frequently discarding functionally usable gadgets increases their expenses. On the other side, a heightened awareness of consequences guides the consumers about their own actions’ impacts on waste accumulation and the severe environmental degradation consequences. This dual awareness should catalyze a reassessment of their consumption pattern, potentially trigger a sense of wastophobia, categorized as a psychological response based on fear of wastefulness.

Second, wastophobia as a psychological force should motivate consumers to change how they use and discard electronic devices, emphasizing the role of community and social considerations. The finding implies that wastophobia, which develops from comprehending spending and their repercussions, makes customers feel guilty. This implies that shame and fear may affect customer behavior, even when involvement is required, like minimizing e-waste. Thus, e-waste disposal education and advertising should incorporate wastophobia to attract customers. Most communications about electronic waste and its effects focus on the negative aspects of societal waste problems, placing consumers at fault. Advocating for social concerns by highlighting personal rewards like greater productivity and boldness in tackling social challenges frequently connects with consumer values, resulting in enough support for the change. Thus, understanding emotional and cognitive factors could help practitioners and legislators build more complete e-waste solutions.

The amplified creative performance, moral courage, and pro-environmental behavior rooted in wastophobia will undoubtedly support the drive toward the minimization of electronic waste and sustainability. To the extent that creative performance is heightened, new products may be developed from old electronics, giving rise to creative repairs and upcycling activities to prolong the lifecycle of the products. Such a trait can also encourage businesses to come up with up and down designs whereby the product is easy to repair or replace some modular parts hence little wastage. In terms of moral courage, it encourages such consumers to support and get involved in recycling programs at their localities, especially those that target e-waste, hence, encouraging the local people to be concerned about waste issues. Furthermore, increased pro-environmental behavior compels consumers to seek eco-friendly goods and services, prompting manufacturers to embrace eco-conscious ways of production to satisfy such needs. All these effects of wastophobia, therefore, transcend the individual level and threaten to revolutionize the management of consumption and production toward a greener future with less electronic waste.

### 5.3 Policy implications

#### 5.3.1 Consumer perspectives

This study provides numerous innovative implications enabling individuals to control their wasteful spending at home considering the importance of wastophobia. First: events like *“Zero Waste Week”* and *“Waste-Free Month,”* should challenge individual households to produce less waste every week or month by giving them incentives to make considerable cuts. This kind of challenging initiatives would help to boost creative performance of each household in community. Second, *“establish Information Sharing Platforms,”* where individuals can share their environmental friendly electronic appliances or gadgets, to boost interest in information sharing regarding waste reduction. By doing this, consumers not only rethink on how to utilize available products optimally but also reduces wastage. Third, introduction of *“digital waste tracking applications.”* Customers may keep track of their waste while also receiving personal tips on how to reduce it making them feel responsible and competitive. Fourth, at last but not least there should be *“Gamification Elements.”* It can enable consumers to earned points through engaging in sustainable consumption practices.

#### 5.3.2 Government perspective

**Figure 6:**
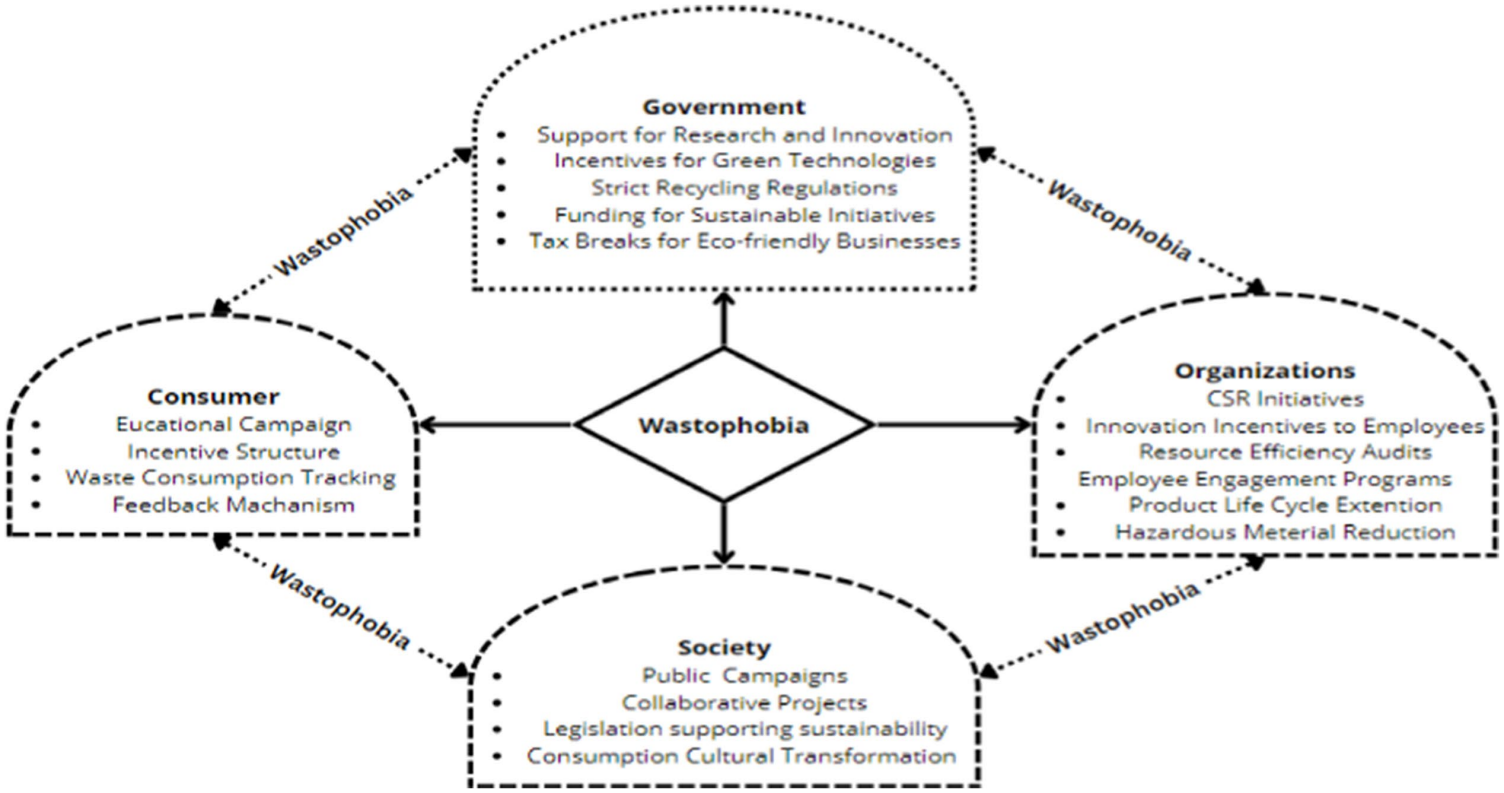
Policy guidelines (Author recommendations).

Government should impose *“stringent recycling regulations”* where consumers and organizations have no option, but to strictly obey waste management policies. Second, *“research and development initiatives”* can be introduce such as *“sustainable green technologies.”* Greener technological initiatives based on extended product lifecycle solution can reduces waste and environmental implications. Moreover, *“financial support and tax breaks”* should be provided to those industries and consumers which care about the environment, might stimulate efforts towards becoming greener. Government can address wastophobia using social media campaigns, co-operation with environmental agencies about recycling and disposal information. By doing this government will instill wastophobia into their customers, thus supporting refuse accumulation and encouraging care for climate (see Figure 6).

#### 5.3.3 Organizational perspective

To guarantee a sustained future, escalated wastophobia should be adopted as an essential pillar of organization’s promotional strategies and firm regulations. Employees who provide sustainable waste-reduction techniques should be provided *“innovation incentives.”* This technique can promote creativity, morality, and sustainability. Second, organization should strictly engage their employees in “*environmental sustainability programs.”* Environmentally conscious employees are more likely to boosts creative skills to redesign new products for extending product life cycles. In addition, companies should focus on *eco-friendly and low carbon materials* instead of poisonous substances like lead and mercury in the manufacturing process. Moreover, organizations need to strengthen the role of *“employees’ customer engagement.”* Sales and marketing staff which directly interact with customers need to escalate wastophobia through information sharing process. Once environmentally conscious consumers understand their wastefulness effects in harming society and the environment, they will prefer to alter their inefficient consumption practices. This feedback approach not only improve organizational loyalty but also can support to increase their profitability.

#### 5.3.4 Societal perspective

The society should formulate a comprehensive set of guidelines for households. Starting with *“community events”* and “*social media campaigns”* a wastophobia culture should be promoted. Wastophobia culture can enhance the understanding of the negative effects of waste on environment and the positive outcomes through consumption reduction tactics. Besides, established wastophobia culture can promote product repair, refurbishing and recycling. This is likely to extend the lifecycle the products of existing products.

### 5.4 Research limitations and future recommendations

The present research has significant results and fills out certain key gaps in the existing literature. Nevertheless, there are still some limitations and research gaps that need to be acknowledged. Initially, the only source of the subjects in the study was the Chinese, therefore the validity of the findings of the study to other cultures and countries is questionable. Therefore, future investigation should evaluate another cultural group of household consumers residing in Asia as well as from the global perspective to fully understand the electronic waste mitigation intentions. Secondly, this study employed a cross-sectional design. Hence, the self-reported scales employed in this research are subject to certain methodological limitations. For instance, to increase their chances in the research, participants may have overestimated their willingness to reduce e-waste which compromised the validity and reliability of the findings (Schmidt, 2016). The following research plans should explore the consideration of tendencies toward wasteful behavior and actual waste reduction behavior in order to enhance generalizability by establishing a connection between intention and manifested behavior. Third, additional significant variables, such as attitudes toward finance, religion, and costs associated with electronic waste, should be included. Further, including aspects like psychographics, total number of people living in a household, family life cycle, as well as income, may also shed some valuable light on the consumer. In addition, we propose that further research should be directed towards consumers who buy electronics items for older parents and relatives as those in authority and respected roles in the family may have a unique perspective on electronic waste behavior.

## 6 Conclusion

The unsustainable production and consumption practices profoundly threaten global initiatives of conserving natural resources, environmental balance, and sustainable economic progress (Roberts et al., 2023). Thus, to control inefficient consumption practices, e-waste, and environmental implications, the present research explores the role of wastophobia, its antecedents, and its consequences’ impact on consumer behavior. A comprehensive model based on the theoretical lenses of the theory of interpersonal behavior and comprehensive model of environmental psychology was developed and tested by obtaining the responses of 302 Chinese household consumers. The current study significantly contributed to the body of knowledge by exploring the potential role of antecedents including awareness of wasteful consumption and awareness of consequences on multiple behavioral outcomes in the presence of wastophobia. The findings have overcome the poor explanatory powers of TPB by suggesting that not only intentions, attitudes, subjective norms, and beliefs supports the change in behavior, but emotional factors like wastophobia can also help to change distorted, irresponsible, inefficient, and unsustainable consumption practices. The findings are consistent with the propositions of TIB and CEMP. Therefore, the study concluded that awareness of wasteful consumption and awareness of consequences are the fundamental antecedents of wastophobia. These factors not only increase consumers’ awareness of their roles in waste production and environmental degradation but also evoke emotions (e.g., guilt, shame, and fear) related to wastefulness. Additionally, wastophobia exerted a chain reaction by activating multiple behavioral consequences including creative performance, moral courage, and a sense of pro-environmental behavior (except consumer advocacy). In other words, consumers living in wastophobia environment can possess a higher degree of wastophobia, and are more likely to engage in e-waste management, extending the usability of product lifecycle, and reducing environmental implications. Such a culture can be achieved through organized public education endorsed by advertising focused on the negative environmental impacts of wasteful acts.

## Data Availability

The raw data supporting the conclusions of this article will be made available by the authors, without undue reservation.

## References

[ref1] Alzaydi A. (2024). Balancing creativity and longevity: the ambiguous role of obsolescence in product design. J. Clean. Prod. 445:141239. doi: 10.1016/j.jclepro.2024.141239

[ref2] Amabile T. M. (1988). A model of creativity and innovation in organizations. Organ. Behav. 10, 123–168.

[ref3] Amabile T. M. (1993). The social psychology of creativity: a componential conceptualization. J. Pers. Soc. Psychol. 45, 357–376. doi: 10.1037/0022-3514.45.2.357, PMID: 40165438

[ref4] Arkorful V. E. Shuliang Z. Lugu B. K. (2023). Investigating household waste separation behavior: the salience of an integrated norm activation model and the theory of planned behavior. J. Environ. Plan. Manag. 66, 2195–2221. doi: 10.1080/09640568.2022.2063112

[ref5] Armstrong R. A. (2019). Should Pearson's correlation coefficient be avoided? Ophthalmic Physiol. Opt. 39, 316–327. doi: 10.1111/opo.12636, PMID: 31423624

[ref6] Arya S. Kumari D. Narzari R. Kumar S. (2023). “A global glance on waste electrical and electronic equipment (WEEEs)” in Global E-waste management strategies and future implications (Elsevier), 1–11. doi: 10.1016/B978-0-323-99919-9.00018-0

[ref8001] Austin P. C. Stuart E. A. (2015). Moving towards best practice when using inverse probability of treatment weighting (IPTW) using the propensity score to estimate causal treatment effects in observational studies. Stat. Med. 34, 3661–3679. doi: 10.1002/sim.6607 26238958 PMC4626409

[ref7] Awang Z. (2012). Structural equation modeling using AMOS graphic: Penerbit Universiti Teknologi MARA. Available at: https://www.scirp.org/reference/ReferencesPapers?ReferenceID=29477466

[ref8] Aydin A. E. Yildirim P. (2021). Understanding food waste behavior: the role of morals, habits and knowledge. J. Clean. Prod. 280:124250. doi: 10.1016/j.jclepro.2020.124250

[ref9] Badawi A. N. Adelazim Ahmed T. S. Alotaibi E. K. Abbas I. S. Ali E. R. Shaker E. S. M. (2024). The role of awareness of consequences in predicting the local tourists’ plastic waste reduction behavioral intention: the extension of planned behavior theory. Sustain. For. 16:436. doi: 10.3390/su16010436

[ref11] Bandura A. (2002). “Social cognitive theory of mass communication” in Media effects: Advances in theory and research. eds. Bryant J. Zillmann D.. 2nd ed (Mahwah, NJ: Taylor & Francis), 121–153.

[ref12] Bravi L. Francioni B. Murmura F. Savelli E. (2020). Factors affecting household food waste among young consumers and actions to prevent it. A comparison among UK, Spain and Italy. Resour. Conserv. Recycl. 153:104586. doi: 10.1016/j.resconrec.2019.104586, PMID: 40348662

[ref13] Brislin R. W. (1986). “The wording and translation of research instruments” in Field methods in cross-cultural research. eds. Lonner W. J. Berry J. W. (Cham: Sage Publications, Inc), 137–164.

[ref14] Brown T. A. (2015). Confirmatory factor analysis for applied research. 2nd Edn. Guilford Publications.

[ref15] Brown M. E. Treviño L. K. (2014). Do role models matter? An investigation of role modeling as an antecedent of perceived ethical leadership. J. Bus. Ethics 122, 587–598. doi: 10.1007/s10551-013-1769-0

[ref7010] Burch R. L. Widman D. R. (2021). The point of nipple erection 1: The experience and projection of perceived emotional states while viewing women with and without erect nipples. Evol. Behav. Sci. 15:305. Available at: https://psycnet.apa.org/record/2020-61355-001

[ref16] Byrne B. M. (2016). Structural equation modeling with AMOS: Basic concepts, applications, and programming. 3rd Edn: Routledge. doi: 10.4324/9780203726532

[ref17] Capoano E. Balbé A. D. Costa P. R. (2024). Is there a “green moral”? How young People’s moral attributes define engagement with narratives about climate change. Soc. Sci. 13:145. doi: 10.3390/socsci13030145

[ref18] Chelminski P. Coulter R. A. (2011). An examination of consumer advocacy and complaining behavior in the context of service failure. J. Serv. Mark. 25, 361–370. doi: 10.1108/08876041111149711

[ref19] Chen Y. T. (2017). The factors affecting electricity consumption and the consumption characteristics in the residential sector – a case example of Taiwan. Sustain. For. 9:1484. doi: 10.3390/su9081484

[ref20] Clayton S. Karazsia B. T. (2020). Development and validation of a measure of climate change anxiety. J. Environ. Psychol. 69:101434. doi: 10.1016/j.jenvp.2020.101434

[ref21] Clydesdale G. (2006). Creativity and competition: the beatles. Creat. Res. J. 18, 129–139. doi: 10.1207/s15326934crj1802_1

[ref22] DeWaters J. Qaqish B. Graham M. Powers S. (2013). Designing an energy literacy questionnaire for middle and high school youth. J. Environ. Educ. 44, 56–78. doi: 10.1080/00958964.2012.682615

[ref23] Dong G. Li R. Li F. Liu Z. Wu H. Xiang L.. (2024). Differences in urban development in China from the perspective of point of interest spatial co-occurrence patterns. ISPRS Int. J. Geo Inf. 13:24. doi: 10.3390/ijgi13010024

[ref7008] Donovan R. (2011). Theoretical models of behaviour change. The SAGE handbook of social marketing, 15–31. Available at: https://www.torrossa.com/en/resources/an/4913738#page=42

[ref24] Edelman (2015). Changing tomorrow’s story, the strategy for energy-efficiency campaign, citizenship report. Fisk, G. (1973), criteria for a theory of responsible consumption. J. Mark. 37:24. doi: 10.2307/1250047, PMID: 39964225

[ref25] Filimonau V. Matute J. Kubal-Czerwińska M. Krzesiwo K. Mika M. (2020). The determinants of consumer engagement in restaurant food waste mitigation in Poland: an exploratory study. J. Clean. Prod. 247:119105. doi: 10.1016/j.jclepro.2019.119105

[ref26] Fisk G. (1973). Criteria for a theory of responsible consumption. J. Mark. 37, 24–31.

[ref27] Gabriel P. J. (2023). A darker side to creativity: How can employees with a fear-of-failure be more creative? Drexel University. ProQuest Dissertations and Theses. Available at: https://search.proquest.com/openview/e6adc010df80811060624bc60b420211/1?pq-origsite=gscholar&cbl=18750&diss=y

[ref7009] Ganti V. Sarma A. D. (2022). Data Cleaning: Springer Nature doi: 10.1007/978-3031=01897-8

[ref28] Ganu J. (2018). Moral courage: the essence of ethical leadership and followership. J. Appl. Christ. Leadership 12, 42–53. Available at: https://digitalcommons.andrews.edu/jacl/vol12/iss2/6

[ref29] González-Rodríguez M. R. Díaz-Fernández M. C. Biagio S. (2019). The perception of socially and environmentally responsible practices based on values and cultural environment from a customer perspective. J. Clean. Prod. 216, 88–98. doi: 10.1016/j.jclepro.2019.01.189

[ref30] Graham-Rowe E. Jessop D. C. Sparks P. (2015). Predicting household food waste reduction using an extended theory of planned behaviour. Resour. Conserv. Recycl. 101, 194–202. doi: 10.1016/j.resconrec.2015.05.020

[ref31] Hair J. Black W. Babin B. Anderson R. (2010). Multivariate data analysis: a global perspective. (Vol. 7th). London: Prentice Hall.

[ref32] Hair J. F. Ringle C. M. Sarstedt M. (2013). Partial least squares structural equation modeling: rigorous applications, better results and higher acceptance. Long Range Plan. 46, 1–12. doi: 10.1016/j.lrp.2013.01.001

[ref7004] Hamby A. Orazi D. Moreau P. (2024). Whose story is this? Source reveal as a communication tactic to increase consumers’ advocacy for social causes. J. Bus. Res. 170:114309. doi: 10.1016/j.jbusres.2023.114309

[ref33] Hanif M. W. Hafeez S. Afridi M. A. (2022). Does wastophobia bring sustainability in consumers’ responsible behavior? A case of electricity waste management. Int. J. Energy Sector Manag. 17, 265–287. doi: 10.1108/IJESM-07-2021-0013

[ref34] Hanif M. W. Hafeez S. Iqbal N. Moshadi Shah S. A. Afridi M. A. (2021). Wastophobia: a path towards sustainability in responsible behavior-a case of domestic sector electricity waste management. Int. J. Energy Econ. Policy 11, 121–129. doi: 10.32479/ijeep.11450

[ref35] Hannah S. T. Avolio B. J. Walumbwa F. O. (2011). Relationships between authentic leadership, moral courage, and ethical and pro-social behaviors. Bus. Ethics Q. 21, 555–578. doi: 10.5840/beq201121436

[ref36] Harland P. Staats H. Wilke H. A. M. (2007). Situational and personality factors as direct or personal norm mediated predictors of pro-environmental behavior: questions derived from norm-activation theory. Basic Appl. Soc. Psychol. 29, 323–334. doi: 10.1080/01973530701665058

[ref39] Ibrahim A. Knox K. Rundle-Thiele S. Arli D. (2018). Segmenting a water use market: theory of interpersonal behavior insights. Soc. Mark. Q. 24, 3–17. doi: 10.1177/1524500417741277

[ref40] Jayasimha K. R. Chaudhary H. Chauhan A. (2017). Investigating consumer advocacy, community usefulness, and brand avoidance. Mark. Intell. Plan. 35, 488–509. doi: 10.1108/MIP-09-2016-0175

[ref41] Karbasi N. S. (2024). Unveiling the potential of perceived authentic leadership to enhance followers’ moral intentions: a self-determination theory perspective. Manag. Res. Rev. 47, 1654–1683. doi: 10.1108/MRR-05-2023-0318

[ref42] Kemper N. S. Campbell D. S. Reiman A. K. (2023). See something, say something? Exploring the gap between real and imagined moral courage. Ethics Behav. 33, 529–550. doi: 10.1080/10508422.2022.2104282

[ref7003] Khan A. D. Saini S. Callen J. (2018). Chronic spontaneous urticaria: Standard management and patient education. This topic last updated. Available at: https://www.uptodate.com/contents/chronic-spontaneous-urticaria-standard-management-and-patient-education

[ref43] Kidder R. M. McLeod B. (2005). Moral courage. New York: W. Morrow.

[ref44] Kline R. B. (2011). Principles and practice of structural equation modeling. 3rd Edn. New York, NY: Guilford Press.

[ref45] Kline T. (2014). An easy guide to factor analysis. Routledge. doi: 10.4135/9781483385693

[ref46] Klockner C. A. (2013). A comprehensive model of the psychology of environmental behaviour—a meta-analysis. Glob. Environ. Chang. 23, 1028–1038. doi: 10.1016/j.gloenvcha.2013.05.014

[ref7012] Kollmuss A. Agyeman J. (2002). Mind the gap: why do people act environmentally and what are the barriers to pro-environmental behavior? Environ. Educ. Res. 8, 239–260. doi: 10.1080/13504620220145401

[ref48] Liu K. Tan Q. Yu J. Wang M. (2023). A global perspective on e-waste recycling. Circul. Econ. 2:100028. doi: 10.1016/j.cec.2023.100028

[ref49] Marks I. M. Mathews A. M. (1979). Brief standard self-rating for phobic patients. Behav. Res. Ther. 17, 263–267. doi: 10.1016/0005-7967(79)90041-X, PMID: 526242

[ref50] Meinel M. Wagner T. F. Baccarella C. V. Voigt K.-I. (2018). Exploring the effects of creativity training on creative performance and creative self-efficacy: evidence from a longitudinal study. J. Creat. Behav. 53, 546–558. doi: 10.1002/jocb.234, PMID: 40351362

[ref51] Miller W. I. (2002). The mystery of courage. Harvard: Harvard University Press.

[ref7001] Mills B. Schleich J. (2010). What’s driving energy efficient appliance label awareness and purchase propensity? Energy Policy 38, 814–825. doi: 10.1016/j.enpol.2009.10.028

[ref52] Mishra P. Pandey C. M. Singh U. Gupta A. Sahu C. Keshri A. (2019). Descriptive statistics and normality tests for statistical data. Ann. Card. Anesthesia 22, 67–72. doi: 10.4103/aca.ACA_157_18, PMID: 30648682 PMC6350423

[ref53] Mumford M. D. Fichtel M. England S. Newbold T. R. (2023). Leader thinking, follower thinking: leader impacts on follower creative performance. Annu. Rev. Organ. Psych. Organ. Behav. 10, 413–440. doi: 10.1146/annurev-orgpsych-120920-045553

[ref54] Mumford M. D. Gustafson S. B. (2007). “Creative thought: cognition and problem solving in a dynamic system” in Creativity research handbook. ed. Runco M. A. (Cresskill, NJ: Hampton), 33–77.

[ref7002] Nawaz M. Abid G. Quartey-Papafio T. K. (2022). Relation of workplace incivility, prosocial motivation and emotional exhaustion to thriving of nurses. Nurs. Res. Rev., 207–222. doi: 10.2147/NRR.S373694

[ref7005] Nawaz M. Zhang P. Hanif M. W. (2025). The Motivators of Sustainable Responisble Consumption behavior: A Sequential mediation. J. Islam. Mark. 14. doi: 10.1108/JIMA-12-2023-0406

[ref55] Ogunfowora B. Maerz A. Varty C. T. (2021). How do leaders foster morally courageous behavior in employees? Leader role modeling, moral ownership, and felt obligation. J. Organ. Behav. 42, 483–503. doi: 10.1002/job.2508

[ref56] Padhy M. K. (2015). Communication through advocacy advertising for public health promotion. Media Watch 6, 92–102. doi: 10.15655/mw/2015/v6i1/55393

[ref7013] Pal S. C. Chakrabortty R. Roy P. Chowdhuri I. Das B. Saha A.. (2021). Changing climate and land use of 21st century influences soil erosion in India. Gondwana Res. 94, 164–185. doi: 10.1016/j.gr.2021.02.021

[ref57] Parvatiyar A. Sheth J. N. (2023). Confronting the deep problem of consumption: why individual responsibility for mindful consumption matters. J. Consum. Aff. 57, 785–820. doi: 10.1111/joca.12534

[ref58] Podsakoff P. M. MacKenzie S. B. Lee J. Y. Podsakoff N. P. (2003). Common method biases in behavioral research: a critical review of the literature and recommended remedies. J. Appl. Psychol. 88, 879–903. doi: 10.1037/0021-9010.88.5.879, PMID: 14516251

[ref62] Roberts T. Hope A. Skelton A. (2017). Why on earth did I buy that? A study of regretted appliance purchases. Philos. Trans. R. Soc. A Math. Phys. Eng. Sci. 375:20160373. doi: 10.1098/rsta.2016.0373, PMID: 28461437 PMC5415650

[ref63] Roberts H. Milios L. Mont O. Dalhammar C. (2023). Product destruction: exploring unsustainable production-consumption systems and appropriate policy responses. Sustain. Product. Consumpt. 35, 300–312. doi: 10.1016/j.spc.2022.11.009

[ref64] Ryan A. M. Spash C. L. (2012). The awareness of consequences scale: an exploration, empirical analysis, and reinterpretation. J. Appl. Soc. Psychol. 42, 2505–2540. doi: 10.1111/j.1559-1816.2012.00951.x

[ref65] Sabokro M. Masud M. M. Kayedian A. (2021). The effect of green human resources management on corporate social responsibility, green psychological climate and employees’ green behavior. J. Clean. Prod. 313:127963. doi: 10.1016/j.jclepro.2021.127963

[ref66] Schultz P. W. (2014). Strategies for promoting pro-environmental behavior. Eur. Psychol. 19, 107–117. doi: 10.1027/1016-9040/a000163

[ref67] Schwartz S. H. (1977). Awareness of consequences and the influence of moral norms on interpersonal behavior. Sociometry, 31:355–369. doi: 10.2307/2786399

[ref68] Sekaran R. B. (2006). *Research methods for business*. A skill building approach. 5th Edn. New York: John Wiley and Sons.

[ref69] Sherif D. M. Abouzid M. Saber A. N. Hassan G. K. (2024). A raising alarm on the current global electronic waste situation through bibliometric analysis, life cycle, and techno-economic assessment: a review. Environ. Sci. Pollut. Res. 31, 40778–40794. doi: 10.1007/s11356-024-33839-0, PMID: 38819510

[ref70] Shimul A. S. Faroque A. R. Teah K. Azim S. M. F. Teah M. (2024). Enhancing consumers' intention to stay in an eco-resort via climate change anxiety and connectedness to nature. J. Clean. Prod. 442:141096. doi: 10.1016/j.jclepro.2024.141096

[ref71] Steinskog D. J. Tjøstheim D. B. Kvamstø N. G. (2007). A cautionary note on the use of the Kolmogorov–Smirnov test for normality. Mon. Weather Rev. 135, 1151–1157. doi: 10.1175/MWR3326.1

[ref72] Subramanian D. Prasad T. B. Sheebapearline B. (2022). A study to identify awareness about green practices in family Celebration-a study with special reference to Tirunelveli District. Neuro Quantology 20:8921. doi: 10.48047/nq.2022.20.11.NQ66890

[ref74] Wang J. He Y. Q. Feng Y. (2024). Analysis and prediction on carbon emissions from electrical and electronic equipment industry in China. Environ. Impact Assess. Rev. 106:107539. doi: 10.1016/j.eiar.2024.107539

[ref75] Wang Y. Wang X. Li M. Dong J. Sun C. Chen G. (2018). Removal of pharmaceutical and personal care products (PPCPs) from municipal waste water with integrated membrane systems, MBR-RO/NF. Int. J. Environ. Res. Public Health 15:269. doi: 10.3390/ijerph15020269, PMID: 29401723 PMC5858338

[ref76] Wolf E. J. Harrington K. M. Clark S. L. Miller M. W. (2013). Sample size requirements for structural equation models: an evaluation of power, bias, and solution propriety. Educ. Psychol. Meas. 73, 913–934. doi: 10.1177/0013164413495237, PMID: 25705052 PMC4334479

[ref78] Yu Z. Gao C. Yang C. Zhang L. (2023). Insight into quantities, flows, and recycling technology of E-waste in China for resource sustainable society. J. Clean. Prod. 393:136222. doi: 10.1016/j.jclepro.2023.136222

[ref79] Zhang Y. Berenguer G. Zhang Z. H. (2024). A subsidized reverse supply chain in the Chinese electronics industry. Omega 122:102937. doi: 10.1016/j.omega.2023.102937

[ref7007] Zhang D. Mushtaque I. Hanif M. W. (2025). Exploring the role of perfectionism and psychological capital in the relationship between academic procrastination, test anxiety and suicidal ideation among pre-medical students. Acta Psychol. 252:104662. doi: 10.1016/j.actpsy.2024.104662 39729815

